# A Logic Gate Based on a Flexible Metal–Organic Framework (JUK‐8) for the Concomitant Detection of Hydrogen and Oxygen

**DOI:** 10.1002/chem.202202255

**Published:** 2022-08-18

**Authors:** Kornel Roztocki, Volodymyr Bon, Irena Senkovska, Dariusz Matoga, Stefan Kaskel

**Affiliations:** ^1^ Inorganic Chemistry I Technische Universität Dresden Bergstrasse 66 01062 Dresden Germany; ^2^ Faculty of Chemistry Adam Mickiewicz University Uniwersytetu Poznańskiego 8 61-614 Poznań Poland; ^3^ Faculty of Chemistry Jagiellonian University Gronostajowa 2 30-387 Kraków Poland

**Keywords:** hydrogen, logic gate, metal–organic frameworks, oxygen, sensors

## Abstract

We present an autonomous, chemical logic gate based on a switchable metal–organic framework (MOF) composite, containing carbon nanoparticles and a Pt catalyst. The switchable MOF composite performs as AND logic gate. Hydrogen and oxygen gas streams serve as binary inputs. Catalytically formed water induces a structural transition (crystal volume expansion) of the MOF, and as a consequence, a detectable drop in conductance of the composite as a ‘true’ output only if both gases come in contact with the composite.

## Graphical abstract

**Boolean MOF Logic**: A switchable metal–organic framework performs AND logic operation using hydrogen and oxygen gas streams as binary inputs.

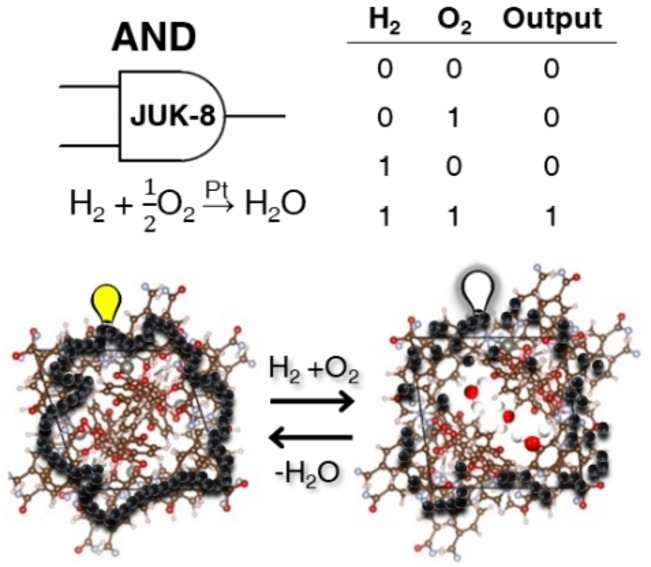

## Introduction

Flexible metal–organic frameworks (MOFs) are a unique class of crystalline porous materials, composed of inorganic nodes connected by organic linkers, that respond to various external physical or chemical stimuli.[^1^, ^2^, ^3^, ^4^, ^5^] Most of these stimuli‐responsive materials, also known as soft porous crystals,^[6]^ directly convert to frameworks with low porosity after solvent molecules removal from their pores. Unlike continuous processes such as thermal expansion/contraction, characteristic of all solids, and swelling, observed for organic polymers,^[7]^ these structural transformations (from the open pore phase, *op* to the closed pore phase, *cp*) occur in a discontinuous (stepwise) manner.[^8^, ^9^, ^10^, ^11^, ^12^, ^13^] The contraction can be reversed by adsorption of fluids. Typically, at a certain pressure/concentration, the MOF structure opens rapidly, significantly increasing the volume of the sample, and the gas diffuses freely into the pores.^[14]^ Depending on the transition mechanism, this adsorption‐induced structural change can be described as breathing or gating.^[5]^

Breathing and gating phenomena rely on the thermodynamic framework bistability.^[4]^ The structural differentiation into phases differing in crystal volume (*op*, *cp*) can be regarded as representing binary information (0,1). Accordingly, flexible MOFs may be applicable for the processing of logic information and the realization of logic gates which are the most elementary building blocks of computing architectures. Semiconductor logic gates rely on transistors and the switching of their electrical conductivity. State of the art complementary metal–oxide–semiconductor (CMOS) technology uses voltage outputs (low, high) to represent binary numbers (0.1) for the realization of Boolean logic gates (AND, NAND, OR, NOR, XOR, NOT etc.) as the most elementary building blocks of integrated circuits.[^15^, ^16^] In chemistry, the first molecular AND logic gate, based on fluorescence signaling, was reported by de Silva et al.^[17]^ and the latest progress was recently summarized.^[18]^ Bistability of flexible frameworks is the basis to develop logical representations based on MOFs for chemical information processing. The concept of logic and symbolism based on flexible MOFs has been rationalized recently.^[19]^ Flexible porous materials are tailor‐made for the response towards fluids rendering them as an ideal platform for realizing logic functions. While many sensor technologies exist for the detection of single gas components,[^20^, ^21^] systems for the concomitant detection of two or more components are rare. However, it is exactly the coincident presence of gases that often causes a high safety risk. In particular, the simultaneous presence of hydrogen and oxygen poses significant occupational safety risks. The transition into a future hydrogen economy requires new technologies to improve the safety of hydrogen transport, storage, and at filling stations.[^22^, ^23^, ^24^, ^25^] Areas requiring the simultaneous detection of two or more components in demanding infrastructures are diverse and include a wide range of explosive gases, for example acetylene, ammonia, ethylene oxide, hydrazine, and silanes to name a few. In biological systems, responsivity towards combined stimuli is an essential feature to guarantee the successful survival of organisms.^[26]^

The integration of flexible MOFs into threshold sensing architectures relies on their structural responsivity accompanied by colossal crystal volume change and the alteration of various physical properties, in particular optical ones.[^27^, ^28^, ^29^, ^30^, ^31^] A highly practical transduction mechanism relies on embedding the crystals into a carbon nanoparticle matrix(CNP).[^32^, ^33^, ^34^] The matrix is conductive as it forms a percolating network. The structural response, however, disrupts the percolation and a stepwise‐change in resistivity enables the detection of gases and vapors, if the critical threshold concentration (gate pressure) is surpassed.

**Scheme 1:**
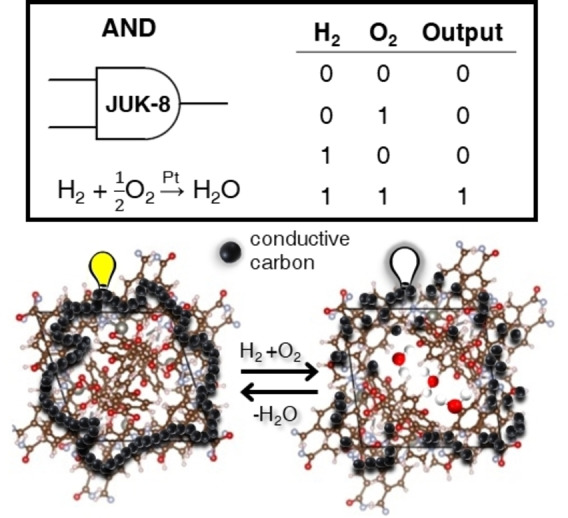
A concept of a chemical AND logic element based on a composite sensing architecture containing switchable framework JUK‐8, conductive carbon nanoparticles, a Vulcan (Pt) catalyst and a binder.

In our proof of concept study, we describe a flexible MOF‐catalyst composite that responds only if two gases are simultaneously present, namely H_2_ and O_2_ representing a logic AND gate (Scheme 1). The framework indicates detection by a crystal volume change relying on a structural gate‐opening mechanism, stimulated by adsorption of water generated in situ on a Pt‐catalyst. Resistance changes of a surrounding carbon nanoparticle matrix serve as a signal. We realize this concept by integrating a water‐stable flexible framework^[34]^ [Zn(oba)(pip)]_n_ (JUK‐8=Jagiellonian University in Kraków‐8; pip=4‐pyridyl functionalized benzene‐1,3‐dicarbohydrazide; oba^2−^=4,4’‐oxydibenzoic carboxylate) into functional devices based on carbon nanoparticles (CNP), Pt‐catalyst (Pt), and polytetrafluoroethylene (PTFE). Under ambient conditions JUK‐8 does not adsorb either H_2_ or O_2_, while it shows a pronounced volumetric expansion of crystals during adsorption of water formed in the reaction between H_2_ and O_2_ on the Pt catalyst. This coincident detection of hydrogen and oxygen by the composite film allows us to realize the first logic operator based on a gating framework material.

## Results and Discussion

**Figure 1:**
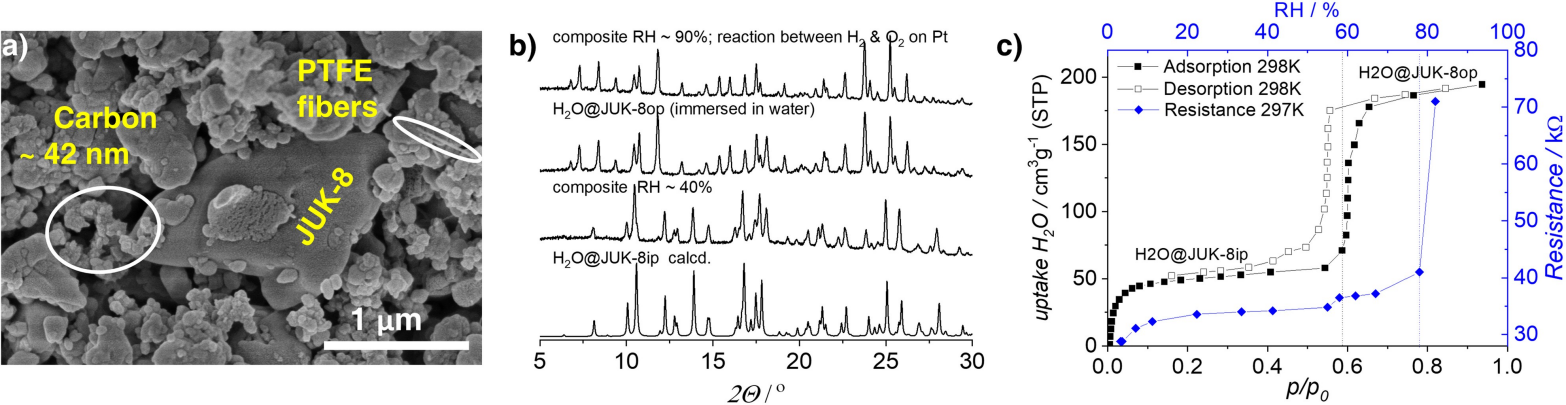
Characteristics of the JUK‐8/CNP/PTFE/Pt composite: a) SEM image showing the distribution of components (before addition of a Pt catalyst). b) PXRD patterns at different humidity levels compared to the PXRD patterns of H_2_O@JUK‐8ip (calculated, based on SC‐XRD data) and H_2_O@JUK‐8op (experimental data). Relative humidity of 90 % was achieved in the reaction between H_2_ and O_2_ on a Vulcan (Pt) catalyst. c) Water physisorption isotherm (at 298 K) for the composite recorded under static conditions (black) compared with the corresponding relationship between the composite film resistance and relative humidity *RH* (blue) measured under dynamic conditions.

The composite consists of a particulate flexible MOF, {[Zn(oba)(pip)] ⋅ 2H_2_O}_n_ (H_2_O@JUK‐8ip) and sieved conductive carbon bound by polytetrafluoroethylene (PTFE). The individual components are easily distinguishable in a scanning electron microscopy (SEM) image (Figure 1a). In our preliminary dynamic experiments, we observed that the repeated adsorption and desorption stress affects the initial resistivity of the composite film, which is related to nanoparticle dislocation and crystal cracking. To eliminate undesirable variability of signals, the composite film attached to a glass slide was preconditioned by wetting with water for 1–2 min which was followed by heating at 100 °C for 30 min. After ten of such immersion and drying cycles, the conductive carbon nanoparticles occupy preferential positions, which prevents them from consecutive relocation in response to repeated adsorption stress. Finally, a layer of Vulcan (20 % Pt) catalyst was placed on top of the composite film. The powder X‐ray diffraction demonstrates that the preparation did not affect the structure of JUK‐8, i. e. at ambient conditions (relative humidity, *RH* ∼40–50 %), the composite includes the H_2_O@JUK‐8ip intermediate phase, which is in agreement with the previous report.^[34]^

Prior to the logic gate experiments, water physisorption measurements were performed on the composite and the data obtained were compared to the relationship between resistance and relative humidity created by the reaction between H_2_ and O_2_ on the surface of the Vulcan (Pt) catalyst (Figure 1c). It is worth noting that the water adsorption isotherm exhibits two steps and each step is associated with a distinct phase transition of JUK‐8. First, the desolvated JUK‐8cp adsorbs two water molecules per zinc already at *RH* ∼6 %, and transforms into the intermediate phase (H_2_O@JUK‐8ip**)**. It causes a small cell volume expansion by 3 % and a hydrogen bonding system stabilizes this structure (Figure S1 and Table S1, Supporting Information), accounting for the first plateau observed in the water isotherm. Then, at *RH* ∼60 %, H_2_O@JUK‐8ip adsorbs another portion of water (eight molecules per zinc) and switches to the {[Zn(oba)(pip)] ⋅ 10H_2_O}_n_ (H_2_O@JUK‐8op) phase. As shown in the previous study, H_2_O@JUK‐8op has about 23 % larger cell volume compared to the closed phase JUK‐8cp. This volume expansion is responsible for the disruption of the surrounding carbon nanoparticle matrix in the composite and its conduction pathway. Under dynamic conditions, the same effect is observed for *RH* ∼80 %, and the difference is due to kinetic limitations. Note that the equilibration time for individual isotherm points is of the order of hours. Furthermore, to demonstrate that JUK‐8 is in the desired open phase at high *RH*, the ex situ PXRD pattern of the composite conditioned at *RH* ∼90 % (created by the active Pt component) was compared with that of JUK‐8 immersed in water (Figure 1b).

**Figure 2:**
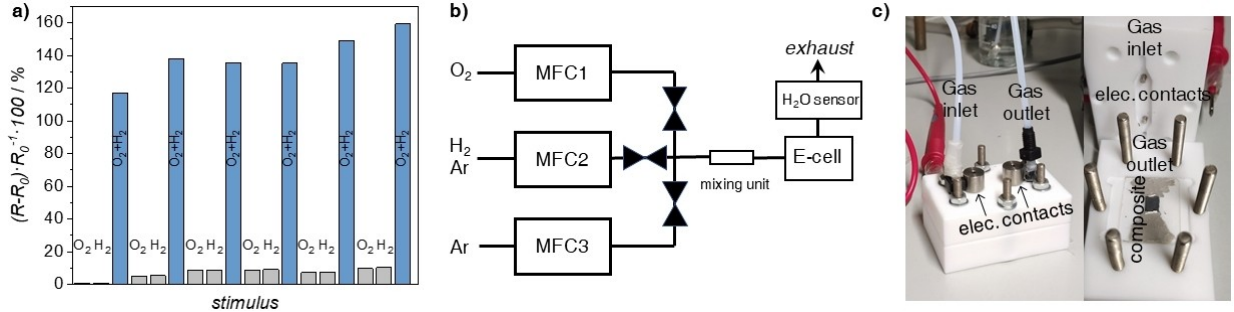
a) Consecutive changes in relative resistance versus a repeated sequence of input gases. b) Schematic drawing and c) optical images of the customized experimental setup used for the experiments. Between the introduction of O_2_ and H_2_ the chamber was flushed by pure Ar for 1–2 minutes. R_0_ – resistance readout for the composite at RH ∼5 % obtained by a 1‐hour dry argon flow. The response time, defined as the interval between the introduction of H_2_ and O_2_ and the maximal signal, is in the range of 10–12 minutes for each cycle. After each cycle, the composite was regenerated in a stream of dry Ar to a steady‐state value (see Table S1, Supporting Information). MFC – mass flow controller. E‐cell – environmental flow cell.

The operation of the logic AND gate is highly reversible demonstrating reproducible resistance changes during several sequential experiments (Figure 2). After the first cycle, the composite was regenerated in a stream of dry argon (Table S2, Supporting Information) and the resistance returned to its initial value (30.2 kΩ). Again, separate dosing of O_2_ or H_2_ had almost no effect on the resistance, *(R‐R_0_)/R_0_* ∼4 %, only the simultaneous presence of H_2_ and O_2_ caused the aforementioned chains of events, leading to a significant increase in resistance*, (R‐R_0_)/R_0_* ∼138 %. Even after six cycles, we observed the reproducibility of the signal, which evidently indicates the working ability of the prepared conductive film. In parallel with the resistance measurements, we measured the response time, that is the time between the introduction of the two gases and the maximum *R* value. For a single cycle, this value is in the range of 10–12 minutes.

Finally, we prepared an analogous MOF/CNP/PTFE/Pt composite (Figure S3, Supporting Information), but instead of the flexible JUK‐8, it included a rigid and stable acylhydrazone‐based MOF, [Cd_2_(sdb)_2_(pcih)_2_]_n_ (sdb^2−^=4,4’‐sulfonyldibenzoic carboxylate; pcih=4‐pyridinecarboxaldehyde hydrazone (pcih).^[14]^ This MOF has both the structure and the water adsorption capacity (230 cm^3^ ⋅ g^−1^) comparable to JUK‐8 (314 cm^3^ ⋅ g^−1^, Figure S4, Supporting Information). The sensor based on the rigid MOF responds to neither O_2_ nor H_2_, nor to the simultaneous presence of H_2_ and O_2_ (Figure S5, Supporting Information). This behavior indicates that water adsorption without simultaneous expansion of the crystal volume has virtually no effect on resistance of the conductive film and the use of a structurally responsive JUK‐8 is crucial to realize the logic gate.

## Conclusion

We realized the first logic gate based on a metal–organic framework using a switchable MOF (JUK‐8) embedded in a composite sensor architecture containing carbon nanoparticles, polytetrafluoroethylene binder and a Pt catalyst (JUK‐8/CNP/PTFE/Pt). The switchable MOF, as a key component of the composite, performs AND logic operation using hydrogen and oxygen gas streams as binary inputs, and provides a ‘true’ output due to its internal structural expansion mechanism and colossal crystal volume change. The Boolean AND response of the MOF is triggered by adsorption of catalytically formed water, resulting in a detectable decrease in conductance of the surrounding matrix of carbon nanoparticles. In this way, the flexible MOF‐based sensing architecture detects the simultaneous presence of hydrogen and oxygen gases. This concept can easily be expanded to a wide range of gaseous species. For example, the simultaneous detection of CO and O_2_ is feasible by gating MOFs responding to CO_2_. In semiconductor industry, BF_3_ and NH_3_ are critical components that could be detected simultaneously by a MOF gate‐opening through the Lewis acid‐base adduct H_3_N‐BF_3_. Furthermore, a remarkable side effect of our development is a “reporting‐function”: Flexible MOFs responding selectively to a product of a chemical reaction “report” if the catalyst is active or not. This concept could have important implications for future in situ technologies for the local detection of catalytic activity in extended industrially used catalyst beds, fuel cell stacks, and academic research.

## Experimental Section

Before the composite fabrication, carbon black and 20 % Pt/Vulcan were sieved using a 200 μm sieve to suppress agglomeration of particles. JUK‐8op was ground in an agate mortar for 4–5 min. 4.6 mg of the sieved CB (5.1 wt %) in 10 mL of MeOH was sonicated for 8–10 min. After that, 80.0 mg of the ground JUK‐8op (89.3 wt %) was added and sonication was continued for further 10 min. The mixture was stirred on a magnetic stirrer at 87 °C (temperature of the heating plate) causing evaporation of the solvent. When the volume of liquid was approximately 3–4 mL, 2 drops of 15 % aqueous suspension of PTFE (approx. 5.0 mg of pure PTFE; 5.6 wt %) was added. After evaporation of the solvent, the resulting solid composite was scraped off and heated for 10 min at 100 °C. The composite was rolled out two times and appropriate film (15×5 mm, ca. 0.10 mm thick) was cut. The composite film was attached to a glass slide by glue (Pattex). The attached composite film was wetted with water and heated at 100 °C for 30 min. After ten such preconditioning cycles, the conductive carbon nanoparticles occupy preferential positions, which prevents them from consecutive relocation in response to repeated adsorption stress. After such treatment, a drop of suspension of 20 % Pt/Vulcan (∼2.5 mg) in MeOH was casted on the composite film. Finally, silver paste was used to make electrical contacts. After that, the composite was heated in an oven for 10 min at 100 °C. The remaining composite was used for PXRD, SEM and water isotherm measurements (at 298 K).

## Conflict of interest

The authors declare no conflict of interest.

1

## Supporting information

As a service to our authors and readers, this journal provides supporting information supplied by the authors. Such materials are peer reviewed and may be re‐organized for online delivery, but are not copy‐edited or typeset. Technical support issues arising from supporting information (other than missing files) should be addressed to the authors.

Supporting Information

## Data Availability

The data that support the findings of this study are available in the supplementary material of this article.
