# Supplementary material for: A Logic Gate Based on a Flexible Metal–Organic Framework (JUK‐8) for the Concomitant Detection of Hydrogen and Oxygen
Source: Chemistry. 2022 Aug 18;28(59):e202202255. doi: 10.1002/chem.202202255 (PMC9804503; doi:10.1002/chem.202202255)
Supplement: Supplementary file 1 — Supporting Information [file CHEM-28-0-s001.pdf]

# Chemistry–A European Journal

Supporting Information

## A Logic Gate Based on a Flexible Metal–Organic Framework (JUK-8) for the Concomitant Detection of Hydrogen and Oxygen

Kornel Roztocki, Volodymyr Bon, Irena Senkovska, Dariusz Matoga, and Stefan Kaskel*

**Table of Contents**

Table of Contents ........ 1
Materials and Methods ........ 1
Figures ........ 3
Tables ........ 6
References ........ 6

**Figure S1** Structural changes induced by water physisorption juxtaposed with the representation of hydrogen bonds (cyan lines) involving water molecules and three **$H_2O$@JUK-8ip** subnetworks. .......... 3
**Figure S2** Experimental setup: gas flow control panel (left), closed environmental chamber (middle) and a composite film inside the environmental chamber (right). ........ 3
**Figure S3** PXRD pattern of a composite based on a rigid MOF, $[Cd_2(sdb)_2(pcih)_2]_n$ (red) compared to a pattern of pristine MOF calculated from the crystal structure (black) compared with water physisorption isotherm (at 298 K) for the composite based on rigid MOF (adsorption - full symbols; desorption - empty symbols). ........ 4
**Figure S4** Water vapor physisorption isotherms for **JUK-8** and $[Cd_2(sdb)_2(pcih)_2]_n$ recorded at 298 K. Data acquired from refs. [1,2]. ........ 4
**Figure S5** Consecutive changes in relative resistance versus the repeating sequence of input gases for conductive composite films based on flexible **JUK-8** (left) and rigid $[Cd_2(sdb)_2(pcih)_2]_n$ (right). Between the introduction of $O_2$ and $H_2$ the chamber was flushed by pure Ar for 1 - 2 minutes. $R_0$ – resistance readout for the composite at RH ~ 5% obtained after a 1-hour dry argon flow. The response time, defined as the interval between the introduction of $H_2$ and $O_2$ and the maximal signal, is in the range of 10 - 12 minutes for each cycle. After each cycle, the composite was regenerated in a stream of dry Ar........... 5
**Figure S6** Tools and materials used to prepare the conductive composite film (left), optical and SEM images of the composite (right). ........ 5

**Table S1** Unit cell volumes (V) and theoretical pore volumes ($V_{pt}$) calculated from single-crystal structures of studied phases of **JUK-8** (Mercury 4.3.1; probe radius 1.3 Å)[3]. ........ 6
**Table S2** Resistance readouts (R) acquired in the experiments. Values are given in kΩ. The reference readout $R_0$ is indicated in bold. ........ 6

## Materials and Methods

**General Remarks.** Unless otherwise stated, all manipulations were done under ambient conditions. All reagents and solvents were of analytical grade (Sigma Aldrich, Merck, POCH, Polmos, Santa Cruz) and were used without further purification. Acetylene Carbon Black (CB) was purchased from ABCR (density 0.2 $g \cdot cm^{-3}$, surface area 64 $m^2 \cdot g^{-1}$, average particle diameter 42 nm).

**Powder X-ray diffraction (PXRD)** PXRD patterns were measured at room temperature on a STOE STADI P diffractometer equipped with Cu-Kα1 radiation ($\lambda$ = 1.54059 Å) and a 1D detector (Mythen,

Dectris). Measurements were performed in transmission geometry using ω/2θ scans and rotating flatbed sample holder.

**Isothermal adsorption analysis**: Water (298 K) adsorption/desorption studies were performed on a BELSORP-max II adsorption apparatus (MicrotracBEL Corp.) Prior to the sorption measurement the composite was degassed in dynamic vacuum at 150 °C for 14 h.

**Scanning electron microscope (SEM)** analysis was performed using a SU8020 scanning electron microscope (HITACHI, Japan) with an accelerating voltage of 1-2 kV. The JUK-8 composite film was sputtered with gold prior to measurements.

**Optical images** were taken by Xiaomi Mi A3.

**Synthesis of MOFs: JUK-8** and $[Cd_2(sdb)_2(pcih)_2]_n$ were prepared according to the published methods[1,2].
Preparation of $\{[Zn(oba)(pip)]\cdot 10H_2O\}_n$ (**$H_2O$@JUK-8op**): $\{[Zn(oba)(pip)]\cdot 2DMF\cdot H_2O\}_n$ (**g@JUK-8@op**) (60 mg) was immersed in 10 mL water for 2 days at room temperature. Crystals were filtered off and the wet material was protected by an X-ray amorphous adhesion tape. Immediately after that, the PXRD pattern was measured at RT.

**Synthesis of MOF/carbon black/PTFE/Vulcan (20% Pt) composite films (Figure S6)**
Before the composite fabrication, carbon black and 20% Pt/Vulcan were sieved using a 200 µm sieve to suppress agglomeration of particles. **JUK-8op** was ground in an agate mortar for 4 - 5 min. 4.6 mg of the sieved CB (5.1 wt %) in 10 ml of MeOH was sonicated for 8-10 min. After that, 80.0 mg of ground **JUK-8op** (89.3 wt.%) was added and sonication was continued for further 10 min. The mixture was stirred on a magnetic stirrer at 87 $^{o}$C (temperature of a plate) causing evaporation of the solvent. When the volume of liquid was approximately 3 - 4 ml, 2 drops of 15% aqueous suspension of PTFE (approx. 5.0 mg of pure PTFE; 5.6 wt.%) was added. After evaporation of the solvent, the resulting solid composite was scraped off and heated for 10 min at 100 $^{o}$C. The composite was rolled out two times and appropriate film (15 x 5 mm, ca. 0.10 mm thick) was cut. The composite film was attached to a glass slide by glue (Pattex). A wetting of the attached composite film with water was performed and heated at 100 $^{o}$C for 30 min. After ten such preconditioning cycles, the conductive carbon nanoparticles occupy preferential positions, which prevents them from consecutive relocation in response to repeated adsorption stress. After that, a drop of suspension of 20% Pt/Vulcan (~2.5 mg) in MeOH was dropped on the composite film. Finally, silver paste was used to make electrical contacts. After that, the composite was heated in an oven for 10 min at 100 $^{o}$C. The remaining composite was used for PXRD, SEM and water isotherm measurements (at 298 K).
The composite based on a rigid MOF $[Cd_2(sdb)_2(pcih)_2]_n$ was prepared in an analogous way.

**Electrical measurement (Figure S2)**
Resistance measurements of the composite film were carried out using a VOLTCRAFT VC-820 digital multimeter. Argon was flushed through a homemade environmental flow chamber at room temperature. The chamber was connected to a hose that split to three inlets, each equipped with a valve and connected to a system of ambient pressure mass flow controllers BRONKHORST (model F-201C-RAD-33V; Fig. 2). The mass flow controllers were further connected to Ar (99.999% purity), $O_2$ (99.5% purity) and $H_2$/Ar ( 5:95 v/v; both 99.999% purity) respectively. Flow rate was 80, 12 and 24 ml/min for Ar, $O_2$ and $H_2$/Ar respectively.

# Figures

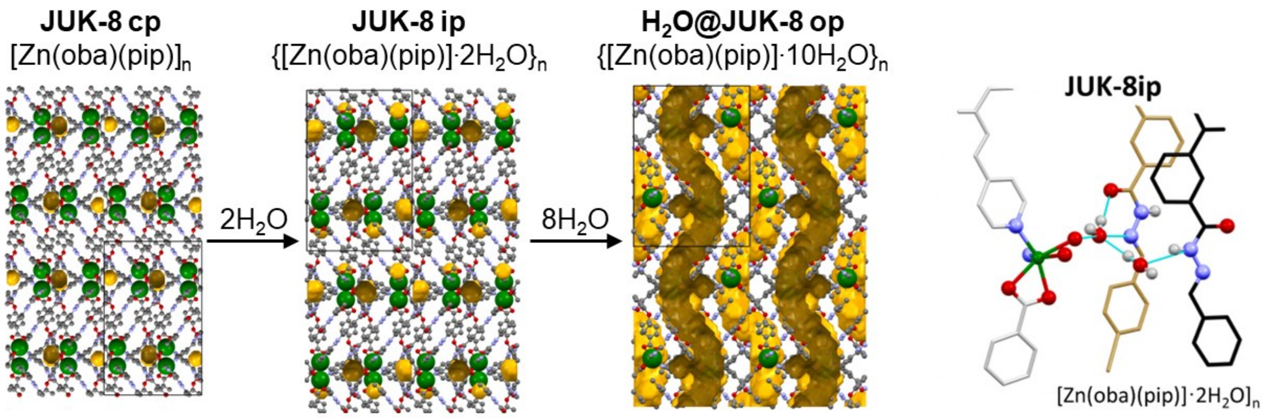


**Figure S1** Structural changes induced by water physisorption juxtaposed with the representation of hydrogen bonds (cyan lines) involving water molecules and three **$H_2O$@JUK-8ip** subnetworks.

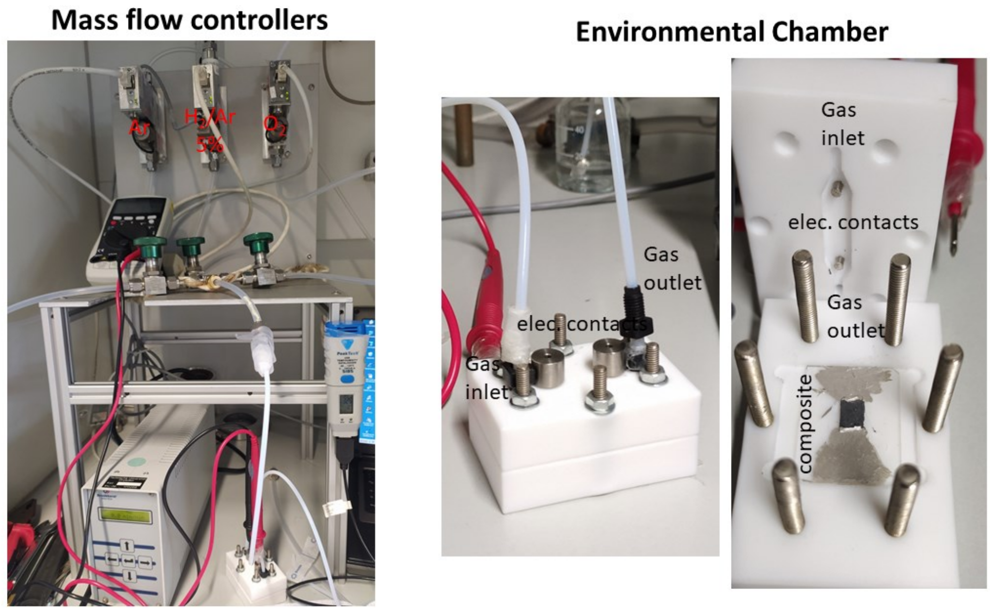


**Figure S2** Experimental setup: gas flow control panel (left), closed environmental chamber (middle) and a composite film inside the environmental chamber (right).

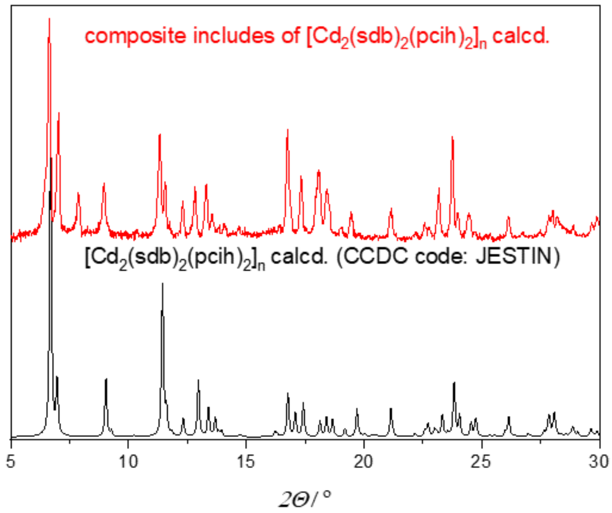


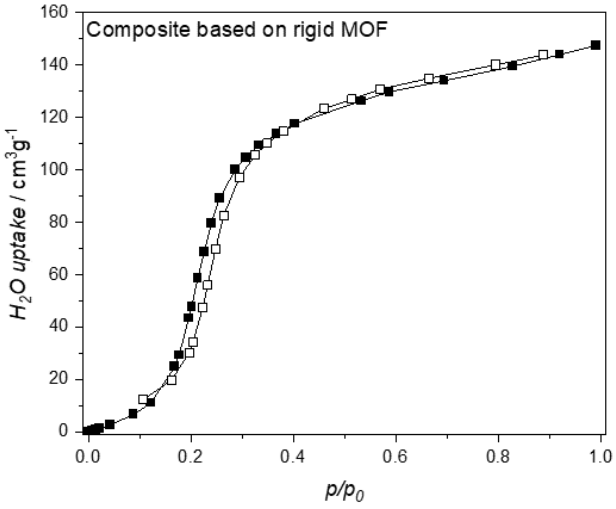


**Figure S3** PXRD pattern of a composite based on a rigid MOF, $[Cd_2(sdb)_2(pcih)_2]_n$ (red) compared to a pattern of pristine MOF calculated from the crystal structure (black) compared with water physisorption isotherm (at 298 K) for the composite based on rigid MOF (adsorption - full symbols; desorption - empty symbols).

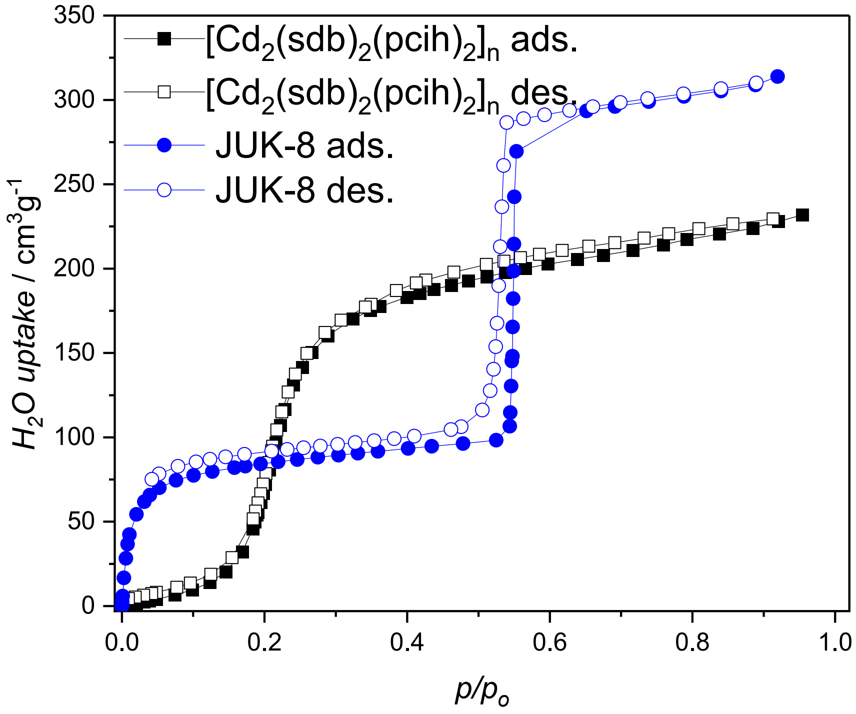


**Figure S4** Water vapor physisorption isotherms for **JUK-8** and $[Cd_2(sdb)_2(pcih)_2]_n$ recorded at 298 K. Data acquired from refs. [1,2].

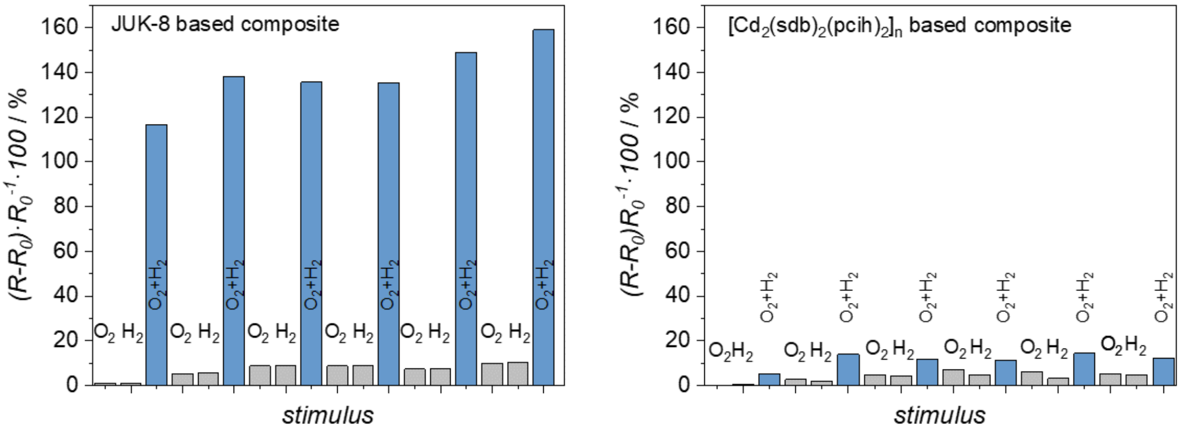


**Figure S5** Consecutive changes in relative resistance versus the repeating sequence of input gases for conductive composite films based on flexible **JUK-8** (left) and rigid $[Cd_2(sdb)_2(pcih)_2]_n$ (right). Between the introduction of $O_2$ and $H_2$ the chamber was flushed by pure Ar for 1 - 2 minutes. $R_0$ – resistance readout for the composite at *RH* ~ 5% obtained after a 1-hour dry argon flow. The response time, defined as the interval between the introduction of $H_2$ and $O_2$ and the maximal signal, is in the range of 10 - 12 minutes for each cycle. After each cycle, the composite was regenerated in a stream of dry Ar.

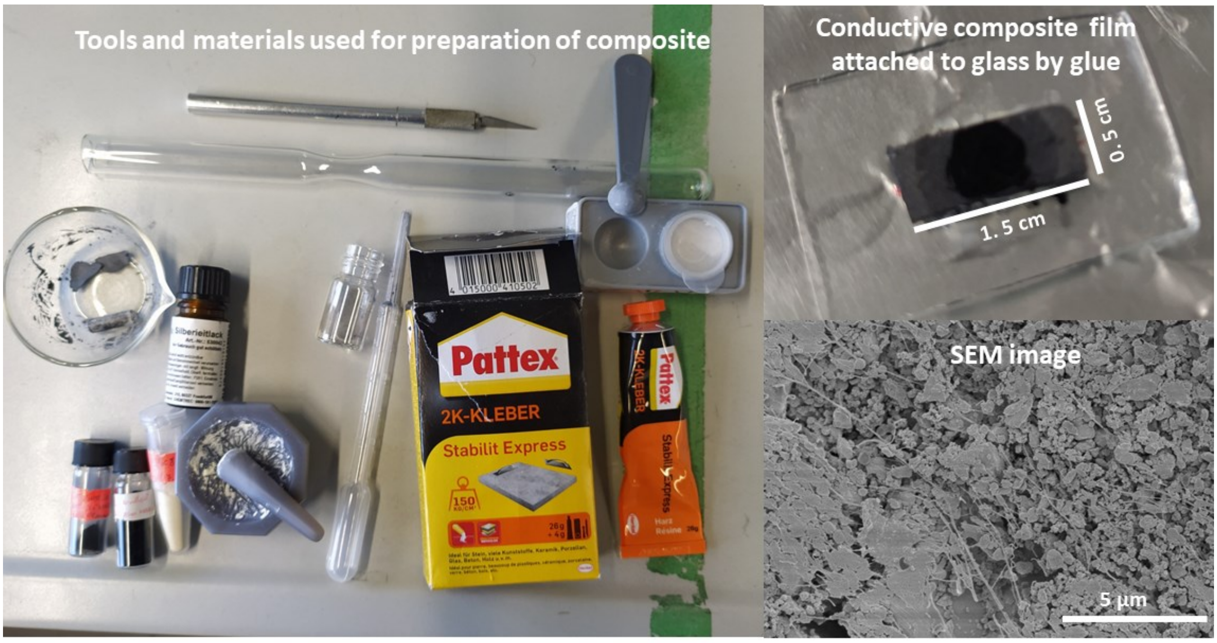


**Figure S6** Tools and materials used to prepare the conductive composite film (left), optical and SEM images of the composite (right).

# Tables

**Table S1** Unit cell volumes (*V*) and theoretical pore volumes ($V_{pt}$) calculated from single-crystal structures of studied phases of **JUK-8** (Mercury 4.3.1; probe radius 1.3 Å)[3].

| | **JUK-8cp** | **$H_2O$@JUK-8ip** | **JUK-8op*** |
|---|---|---|---|
| $V$ /Å$^3$ | 6531 | 6727 | 8050 |
| $V_{pt}$ / cm$^3$/g | 0.012 | 0.035 | 0.241 |

*data acquired for the phase containing DMF and water molecules, however the geometrical calculation indicates that the pore volume of this phase is comparable to **$H_2O$@JUK-8op**

**Table S2** Resistance readouts (*R*) acquired in the experiments. Values are given in kΩ. The reference readout $R_0$ is indicated in bold.

| **Cycle** | | | | | | | | | | | |
|---|---|---|---|---|---|---|---|---|---|---|---|
| **1st** | | **2nd** | | **3rd** | | **4th** | | **5th** | | **6th** | |
| **Ar ($R_0$)** | **28.6** | Ar | 30.2 | Ar | 31.4 | Ar | 31.2 | Ar | 30.7 | Ar | 31.4 |
| $O_2$ | 28.9 | $O_2$ | 30.1 | $O_2$ | 31.1 | $O_2$ | 31.1 | $O_2$ | 30.7 | $O_2$ | 31.6 |
| Ar | 28.8 | Ar | 30.1 | Ar | 31.1 | Ar | 31.1 | Ar | 30.7 | Ar | 31.4 |
| $H_2$ | 28.9 | $H_2$ | 30.2 | $H_2$ | 31.1 | $H_2$ | 31.2 | $H_2$ | 30.8 | $H_2$ | 31.4 |
| $H_2$ + $O_2$ | 62.2 | $H_2$ + $O_2$ | 68.1 | $H_2$ + $O_2$ | 67.4 | $H_2$ + $O_2$ | 67.3 | $H_2$ + $O_2$ | 71.2 | $H_2$ + $O_2$ | 74.1 |

## References

[1] K. Roztocki, F. Formalik, A. Krawczuk, I. Senkovska, B. Kuchta, S. Kaskel, D. Matoga, *Angew. Chem. Int. Ed.* **2020**, *59*, 4491–4497.

[2] K. Roztocki, M. Lupa, A. Sławek, W. Makowski, I. Senkovska, S. Kaskel, D. Matoga, *Inorg. Chem.* **2018**, *57*, 3287–3296.

[3] K. Roztocki, M. Rauche, V. Bon, S. Kaskel, E. Brunner, D. Matoga, *ACS Appl. Mater. Interfaces* **2021**, *13*, 28503–28513.
